# Association between betatrophin/ANGPTL8 and non-alcoholic fatty liver disease: animal and human studies

**DOI:** 10.1038/srep24013

**Published:** 2016-04-05

**Authors:** Yong-ho Lee, Sang-Guk Lee, Chan Joo Lee, Soo Hyun Kim, Young-Mi Song, Mi Ra Yoon, Byung Hun Jeon, Jae Hyuk Lee, Byung-Wan Lee, Eun Seok Kang, Hyun Chul Lee, Bong-Soo Cha

**Affiliations:** 1 Department of Internal Medicine, Yonsei University College of Medicine, Seoul, Republic of Korea; 2 Department of Laboratory Medicine, Yonsei University College of Medicine, Seoul, Republic of Korea; 3 Division of Cardiology, Severance Cardiovascular Hospital and Cardiovascular Research Institute, Yonsei University College of Medicine, Seoul, Republic of Korea; 4 Brain Korea 21 PLUS Project for Medical Science, Yonsei University College of Medicine, Seoul, Republic of Korea; 5 Division of Endocrinology, Department of Internal Medicine, Seonam University College of Medicine, Goyang-si, Gyeonggi-do, Republic of Korea

## Abstract

Betatrophin/angiopoietin-like protein 8 (ANGPTL8) is a liver-secreted protein recently identified as a potent stimulator of beta cell proliferation in mice. However, it is unclear how betatrophin is regulated in humans with non-alcoholic fatty liver disease (NAFLD). We investigated the role of betatrophin in mice and in humans with and without NAFLD. Serum betatrophin levels were examined by ELISA in 164 subjects, including 96 patients with NAFLD. Levels were significantly elevated in subjects with NAFLD compared with controls (1.301 ± 0.617 vs. 0.900 ± 0.574 μg/L, P < 0.001), even after stratification by diabetic or obesity status. Circulating betatrophin positively correlated with obesity or glycemic indices, liver enzyme profiles, and NAFLD status, and was confirmed by multivariate regression analyses (β = 0.195, P = 0.040). However, when including insulin resistance index in the model, the significant association between betatrophin level and NAFLD was diminished due to a mediation effect of insulin resistance on this relationship. Palmitate or tunicamycin increased betatrophin expression in HepG2 cells, while a chemical chaperone blocked its induction. Hepatic expression of betatrophin was elevated in mice with NAFLD including db/db or ob/ob mice and mice with a high-fat or methionine-choline deficient diet. In conclusion, circulating betatrophin was increased in mice and humans with NAFLD and its expression was induced by endoplasmic reticulum stress in hepatocytes (Clinical trial no. NCT02285218).

Non-alcoholic fatty liver disease (NAFLD) is emerging as a worldwide public health issue because the prevalence of obesity is increasing, populations are aging, and sedentary lifestyles prevail^1^. NAFLD is defined histologically as the accumulation of ectopic liver fat (mainly triglycerides) in more than 5% of hepatocytes with no evidence of significant alcohol intake or other secondary etiology of liver disease. It is implicated in an increased risk of mortality^2^ as well as serious comorbidities like diabetes and cardiovascular diseases^3^,^4^,^5^. Among subgroups of NAFLD, non-alcoholic steatohepatitis (NASH) is a progressive and serious condition which can further develop into cirrhosis or hepatocellular carcinoma^6^,^7^. Compared to simple steatosis, NASH has greater risks of liver-related or cardiometabolic complications^5^. Despite its high prevalence and consequences, NAFLD screening and detection primarily depends on expensive imaging devices, including ultrasonography, or blood assays of liver enzyme profiles, which are often inaccurate^8^. This conundrum demonstrates a critical need for reliable biomarkers to identify NAFLD.

Recently betatrophin, also termed angiopoietin-like protein 8 (ANGPTL8), lipasin, or Re-feeding Induced in Fat and Liver (RIFL), has been introduced as a novel protein that is secreted primarily from fat and the liver in mice and is likely to modulate glucose homeostasis and lipid metabolism^9^. Betatrophin knockout mice have decreased serum levels of triglycerides^10^,^11^, whereas betatrophin overexpression has been shown to markedly elevate serum triglycerides^12^,^13^. These findings are consistent with previous evidence of the inhibitory function of betatrophin on lipoprotein lipase activity^10^. Yi *et al.* showed that mice with severe insulin resistance have a dramatically increased level of betatrophin, which could induce pancreatic β-cell proliferation and lower blood glucose levels^14^.

Contrary to several animal studies showing that liver and brown/white fat are major betatrophin-producing tissues^12^,^13^,^15^, in humans betatrophin is highly expressed mainly in the liver^12^, suggesting a species-specific expression pattern. Recent human studies reported that serum betatrophin levels are increased in subjects with type 1 diabetes (T1D)^16^, obesity^17^, and type 2 diabetes (T2D)^17^,^18^,^19^, However, there is no report of the role of betatrophin in patients with fatty liver. Considering that obesity, T2D, and NAFLD share insulin resistance as a common pathophysiologic mechanism^20^ and that the dominant expression of betatrophin is in human liver^12^, we hypothesized that circulating betatrophin levels might be elevated in subjects with NAFLD.

Therefore, the present study investigates the circulating levels of betatrophin and the clinical parameters associated with betatrophin levels in subjects from a human cohort with or without NAFLD, which is further elucidated using *in vivo* and *in vitro* models.

## Results

### Baseline characteristics of the study population

Baseline characteristics of study participants according to NAFLD status are shown in Table 1. Among a total of 164 subjects (30 normal subjects, 25 subjects with impaired fasting glucose [IFG], and 109 subjects with T2D), 134 age- and sex-matched patients were examined by imaging devices to assess hepatic steatosis, which resulted in 96 (72%) of subjects classified as having NAFLD. Subjects with NAFLD had a significantly higher body mass index (BMI) and serum levels of fasting glucose, glycosylated hemoglobin (HbA1c), alanine aminotransferase (ALT), aspartate transaminase (AST), triglyceride (TG), fasting insulin, and homeostasis model assessment of insulin resistance (HOMA-IR), compared to those without NAFLD. A higher number of patients with T2D were included in the NAFLD group; there was no significant difference in other parameters, such as age, blood pressure, total cholesterol (TC), and low-density lipoprotein cholesterol (LDL-C), between the two groups.

### Elevated betatrophin levels in human subjects with non-alcoholic fatty liver disease

**Figure 1:**
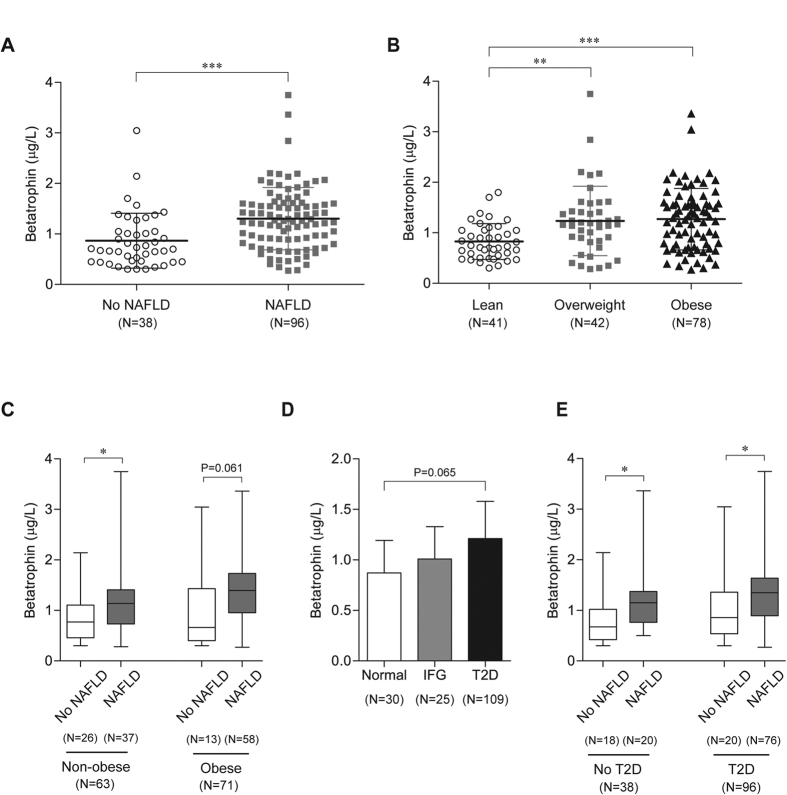
Comparison of serum betatrophin levels in subjects according to the status of NAFLD, obesity, and diabetes. (**A**) Serum betatrophin levels were elevated in NAFLD patients. (**B**) Serum betatrophin levels are increased in subjects who were overweight and obese. (**C**) Box-and-whisker plots of betatrophin levels in subjects with and without NAFLD, stratified by their obesity status. Box indicates first and third quartiles and median values with whiskers from minimum to maximum. (**D**) Comparison of circulating betatrophin levels among normal, IFG, and diabetic subjects. Data are expressed as mean ± SD. (**E**) Box-and-whisker plots of betatrophin levels in subjects with and without NAFLD, stratified by their glycemic condition. Box indicates first and third quartiles and median values with whiskers from minimum to maximum. *P < 0.05; **P < 0.005 and ***P < 0.001.

Serum betatrophin levels were significantly elevated in subjects with NAFLD compared with controls (1.301 ± 0.617 vs. 0.900 ± 0.574 μg/L, P < 0.001, Fig. 1A). Obese and overweight individuals also showed increased levels of betatrophin (1.271 ± 0.608 vs. 1.234 ± 0.686 vs. 0.828 ± 0.356 μg/L, P < 0.001, Fig. 1B). Furthermore, non-obese subjects with NAFLD had higher serum betatrophin concentrations (1.188 ± 0.467 vs. 0.849 ± 0.467 μg/L, P = 0.030, Fig. 1C), whereas obese subjects with NAFLD showed a marginal increase in betatrophin levels (1.367 ± 0.579 vs. 0.979 ± 0.799 μg/L, P = 0.061). Similar to recent reports showing that betatrophin levels were increased in subjects with T2D or insulin resistance^17^,^18^,^19^, serum betatrophin levels were mildly elevated, with no statistically significant difference between subjects with IFG or T2D compared to normal subjects (P = 0.065, Fig. 1D). After stratification by glycemic status, we demonstrated that serum betatrophin levels were significantly elevated in NAFLD subjects regardless of the presence of T2D (no T2D group: 1.197 ± 0.638 vs. 0.797 ± 0.506 μg/L, P = 0.041; T2D group: 1.328 ± 0.613 vs. 0.992 ± 0.627, P = 0.032; Fig. 1E).

### Association of betatrophin with clinical parameters

Next, we evaluated the association between serum betatrophin levels and various clinical and biochemical profiles (Table 2). In the Spearman correlation analysis, betatrophin levels had strong positive correlations with HbA1c (*r* = 0.439), NAFLD status (*r* = 0.330), ALT (*r* = 0.326), HOMA-IR (*r* = 0.320), and fasting glucose (*r* = 0.310), respectively. BMI (*r* = 0.281), fasting insulin (*r* = 0.255), AST (*r* = 0.226), and TG levels (*r* = 0.241) were also significantly related to betatrophin levels.

To adjust for age, sex and other covariates, which were significantly associated with betatrophin levels by Spearman correlation, multivariate regression analyses were conducted (Table 2). In model 1, in which AST and ALT levels were excluded, betatrophin levels were independently associated with HbA1c (STD β = 0.410, P = 0.008) and NAFLD (STD β = 0.204, P = 0.028). In model 2, after adding the liver enzyme profiles, these associations were not significantly altered. However, in model 3, which included HOMA-IR (fasting glucose was removed from the model due to collinearity), HbA1c remained statistically significantly associated with betatrophin levels (STD β = 0.300, P = 0.005), whereas the association between NAFLD and betatrophin levels was diminished in this model. This finding from model 3 can be explained by the mediation effect of HOMA-IR on the relationship between betatrophin and NAFLD (average causal mediation effect = 0.074, 95% confidence interval = 0.016–0.148, P = 0.040).

**Figure 2:**
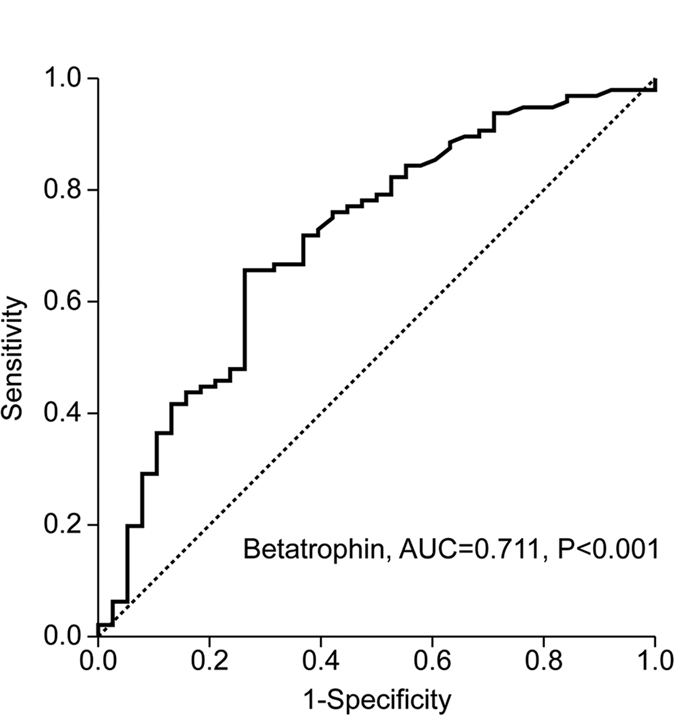
A ROC curve with serum betatrophin levels for the prediction of NAFLD.

Figure 2 presents the receiver operating characteristic (ROC) curve of serum betatrophin levels to determine the utility of this biomarker for predicting NAFLD. The area under the ROC curve (AUROC) was 0.711 (95% confidence interval =0.613–0.810, P < 0.001). Use of betatrophin cutoff of 1.051 μg/L was associated with the highest value to predict NAFLD with regard to the following: the Youden Index 39; sensitivity 65.6%; specificity 73.7%.

### Tunicamycin-induced ER stress increased the expression of betatrophin

**Figure 3:**
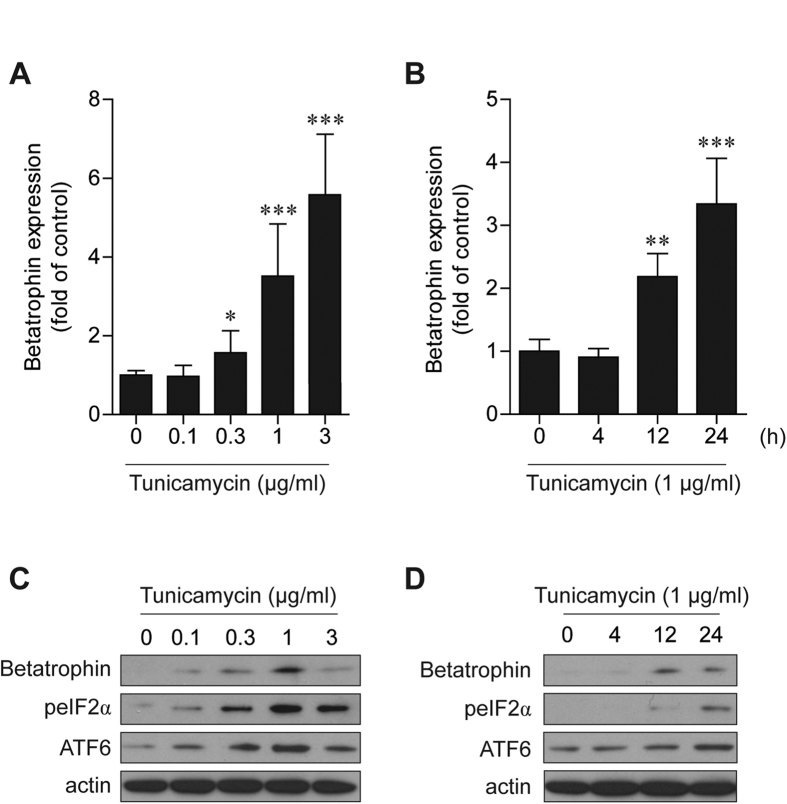
Tunicamycin-induced ER stress increased betatrophin expression. (**A**,**B**) Tunicamycin increased betatrophin mRNA expression in HepG2 cells in a dose- (**A**) and time (**B**)-dependent manner. (**C**,**D**) Tunicamycin increased betatrophin and ER stress marker protein expression in HepG2 cells in a dose- (**C**) and time- (**D**) dependent manner. Cells were treated with various concentrations of tunicamycin for 12 h prior to quantitative real-time PCR and 24 h prior to immunoblots. For the time-dependent experiment, 1 μg/ml of tunicamycin was added. Data are expressed as mean ± SD obtained from at least six individual experiments. *P < 0.05; **P < 0.01; ***P < 0.001 compared with controls.

Endoplasmic reticulum (ER) stress plays a primary role in NAFLD development and progression to non-alcoholic steatohepatitis^21^. Therefore, tunicamycin, an ER stress inducer, was used to investigate whether betatrophin expression is regulated by ER stress. Tunicamycin treatment increased betatrophin mRNA levels in HepG2 cells in both a dose- and time-dependent manner (Fig. 3A,B). Elevated betatrophin protein expression was also confirmed with immunoblots (Fig. 3C,D). Tunicamycin significantly activated ER stress markers such as eIF2α and ATF6, in HepG2 cells.

### Inhibition of ER stress suppressed tunicamycin or palmitate-induced expression of betatrophin

**Figure 4:**
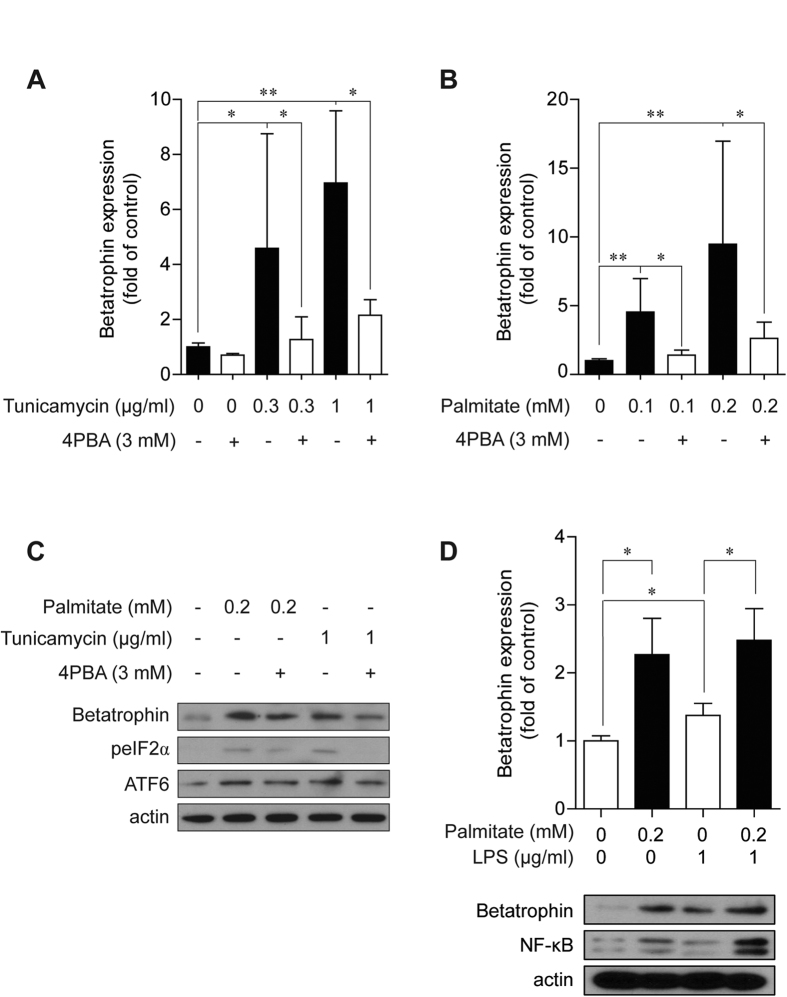
ER stress inhibition by 4-PBA, a chemical chaperone, suppressed palmitate- and tunicamycin-induced betatrophin expression. (**A**,**B**) Tunicamycin (**A**) and palmitate (**B**) increased betatrophin mRNA expression in HepG2 cells in a dose-dependent manner and its effect was suppressed by 4-PBA pretreatment. (**C**) Tunicamycin and palmitate induced betatrophin and ER stress marker protein expression in HepG2 cells. Pretreatment with 3 mM PBA significantly decreased betatrophin expression. (**D**) Betatrophin mRNA expression in HepG2 cells was increased with LPS treatment, but was not additionally induced by combined treatment with palmitate and LPS. Immunoblots revealed that betatrophin and NF-κB were induced by LPS treatment. Cell lysates were harvested after 12 or 24 h of treatment for betatrophin detection using quantitative real-time PCR or immunoblots, respectively. Data are expressed as mean ± SD obtained from at least six individual experiments. *P < 0.05 and **P < 0.01 compared with controls.

To further confirm the role of ER stress in the expression of betatrophin, a chemical chaperone, 4-phenylbutyric acid (4-PBA), was used as an ER stress inhibitor. Tunicamycin-induced mRNA expression of betatrophin (Fig. 4A) was significantly attenuated by 4-PBA (3 mM), indicating involvement of ER stress in betatrophin expression regulation. Next we investigated whether the hepatic betatrophin expression is induced by palmitate, which aggravates NAFLD and is one of the most abundant saturated free fatty acids in plasma. Similar to tunicamycin, palmitate elevated betatrophin mRNA levels in HepG2 cells (Fig. 4B), which were also attenuated by pretreatment with 4-PBA. We confirmed that 4-PBA effectively inhibited tunicamycin- or palmitate-induced expression of ER stress markers such as eIF2α and ATF6 as well as betatrophin protein in HepG2 cells (Fig. 4C). To evaluate whether immune stress signaling is involved in betatrophin expression, HepG2 cells were treated with lipopolysaccharides (LPS) to stimulate toll-like receptors (TLRs). LPS alone did significantly increase the expression of betatrophin, while combined treatment with LPS and palmitate did not additionally increase betatrophin mRNA in HepG2 cells (Fig. 4D). Immunoblots confirmed that betatrophin and NF-κB were induced by treatment with LPS.

### Betatrophin expression is increased in various animal models with fatty liver

**Figure 5:**
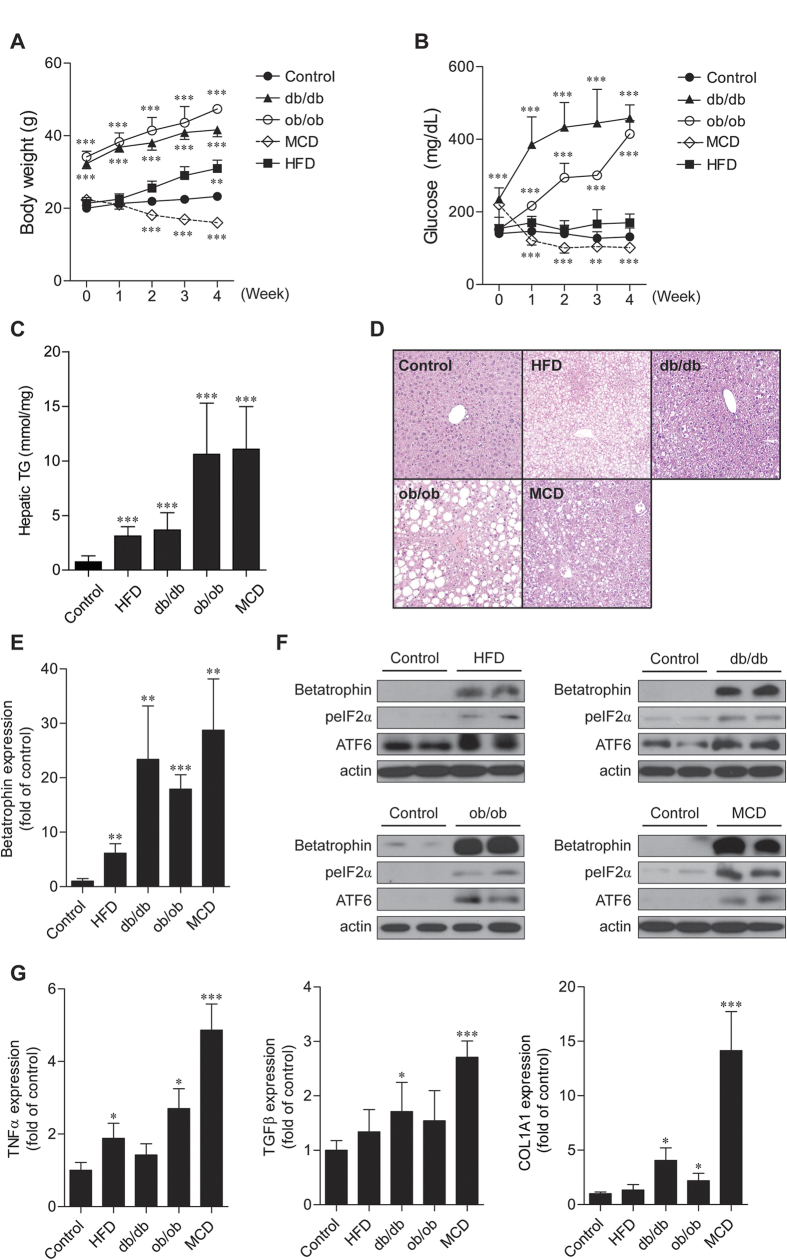
Betatrophin expression in various fatty liver animal models. (**A**,**B**) Changes in body weights (**A**) and serum glucose levels (**B**) in control, db/db and ob/ob mice, and mice fed with high-fat diet (HFD) and methionine-choline-deficient (MCD) diet. The x axis represents 2, 4, 6, and 8 weeks for the HFD group. Black circles = control mice; black triangles = db/db mice; white circles = ob/ob mice; black squares = mice fed with HFD; white diamonds = mice fed with MCD. (**C**,**D**) Hepatic TG contents (**C**) and liver histology ((**D**,**H**,**E**) stain) in control, high-fat diet (HFD)-fed, db/db, ob/ob, and MCD-fed mice. Magnification, 200×. (**E**) The hepatic betatrophin mRNA expression in the various animal models. (**F**) Hepatic betatrophin and ER stress marker protein expression in various animal models. (**G**) mRNA expression of inflammation and fibrosis-related genes in various animal models. Data are expressed as mean ± SD (**A**,**B**,**C**) or SEM (**E**,**G**) obtained from at least six individual experiments. *P < 0.05; **P < 0.01; ***P < 0.001 compared with control mice.

To investigate whether betatrophin expression is increased in mice with fatty liver *in vivo*, we used db/db, ob/ob mice, and mice treated with an high fat diet (HFD) or methionine-choline deficient (MCD) diet. Changes in body weight and serum glucose levels were elevated in db/db, ob/ob, and HFD-induced obese mice, but were significantly decreased in MCD diet-fed mice compared with control C57BL6J mice (Fig. 5A,B). Hepatic TG contents were remarkably increased in db/db, ob/ob, HFD, and MCD diet-fed mice compared with controls (Fig. 5C). Hepatic lipid droplets were accumulated in these mouse models (Fig. 5D). The hepatic expression of betatrophin mRNA (Fig. 5E) and protein (Fig. 5F) was significantly elevated in db/db, ob/ob, HFD, or MCD diet-fed mice compared with controls. Increased liver levels of ER stress markers were also confirmed in these mice (Fig. 5F). Furthermore, expression of inflammation and fibrosis-related genes was also increased in these models (Fig. 5G). Notably, the strongest expression of TNFα, type Iα collagen (COL1A1), and TGFβ in MCD mice correlated with the highest expression of betatrophin.

**Figure 6:**
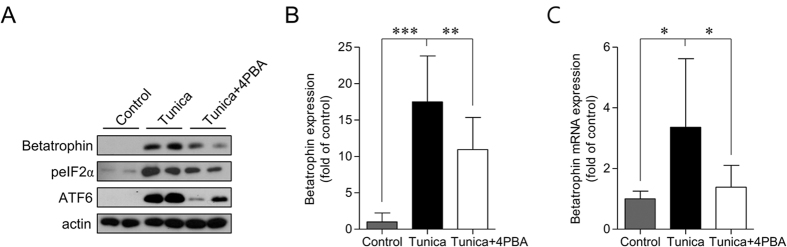
Administration of tunicamycin significantly elevated hepatic expression of betatrophin in mice, while its expression was diminished by the pre-treatment of 4-PBA. Liver lysates were harvested after 24 h of treatment with tunicamycin for protein or mRNA detection using immunoblots (**A**,**B**) or quantitative real-time PCR (**C**), respectively. (**B**) Densitometric graph of the optical density-based data of the immunoblots. Data are expressed as mean ± SD. **P < 0.01 and ***P < 0.001.

Tunicamycin-treated mice were used to confirm the role of ER stress in the regulation of betatrophin expression *in vivo*. Tunicamycin markedly increased hepatic expression levels of betatrophin as well as ER stress markers as compared to the control group (Fig. 6A,B). Pre-treatment with ER stress inhibitor, 4-PBA significantly decreased betatrophin expression in the liver (Fig. 6C).

## Discussion

The present study demonstrated for the first time, that serum betatrophin levels were significantly elevated in patients with NAFLD compared with controls. This association was maintained even after controlling for diabetic status. In addition, serum betatrophin concentration was positively correlated with glycemic and liver-related laboratory parameters as well as TG or HOMA-IR levels. Furthermore, the human study results were supported by animal experiments *in vivo*. These experiments showed that various fatty liver mouse models had markedly increased hepatic betatrophin expression. Both pharmacologically- and nutritionally-activated ER stress and TLR signaling induced betatrophin expression in hepatocytes *in vitro*.

Betatrophin/ANGPTL8 is a recently identified, novel protein secreted from hepatic or adipose tissues. It has been proposed to play a dual role in TG metabolism and pancreatic β-cell proliferation by independent research groups^9^,^12^,^13^,^22^. Quagliarini *et al.* suggested that ANGPTL8 regulates postprandial TG and fatty acid levels by inhibiting the lipoprotein lipase through interaction with ANGPTL3^22^. A mouse model of severe insulin resistance showed a markedly increased betatrophin level, which acted as a potent mitogen for insulin-producing β-cells^14^. However, a subsequent study reported contradictory results, demonstrating no impairment in β-cell expansion in betatrophin knockout mice^23^. These conflicting results raised controversy regarding the function of betatrophin in relation to glucose metabolism. In addition, Wang *et al.* clearly showed that betatrophin knockout mice manifested lower levels of serum TG with reduced fat mass, but no alteration in glucose homeostasis^10^, indicating that betatrophin may be required for lipid metabolism regulation. A recent study proposed that betatrophin modulates the autophagic process, which leads to regulation of hepatic lipid turnover^24^.

With regard to the tissue expression pattern of betatrophin, betatrophin mRNA was mainly expressed in the liver and in brown and white adipose tissues in mice^12^,^13^,^14^,^15^. However, in humans it is predominantly enriched in the liver^12^, suggesting that betatrophin might serve as a hepatokine for which the function remains to be fully established. Based on this finding, we focused on betatrophin expression regulation in a human hepatocyte cell line. To date, there is no report regarding betatrophin expression distribution in other human tissues.

With regard to betatrophin expression regulation, nutritional status (fasting and feeding) can effectively modulate mRNA levels in mice livers^13^ as well as in brown and white fat tissues^12^,^15^. In 3T3-L1 adipocytes, betatrophin expression was markedly increased during adipogenesis and was induced by insulin treatment^13^. However, tumor necrosis factor α or peroxisome proliferator-activated receptor γ knockdown significantly suppressed the betatrophin expression in 3T3-L1 adipocytes^13^. The present study showed that hepatic betatrophin expression was significantly elevated in mouse models with fatty liver, such as db/db, ob/ob, and HFD or MCD diet-fed mice. Consistent with our findings, both epididymal fat and liver showed increased betatrophin mRNA levels in ob/ob mice^13^, In addition, hepatic betatrophin expression was upregulated in sterol regulatory element-binding protein (SREBP)-1a or SREBP-2 transgenic mice and mice treated with a liver X receptor agonist^22^, all of which displayed hepatic steatosis^25^,^26^. The association between betatrophin and NAFLD is also supported by the recent study showing that hepatic lipid droplets were surrounded by intracellular betatrophin in hepatocytes^24^.

Notably, our study demonstrated that palmitate- or tunicamycin-mediated ER stress induced betatrophin expression in hepatocytes. It is well established that hyperlipidemia or lipotoxicity can activate ER stress, which then contributes to the development of insulin resistance, T2D, and NAFLD^27^,^28^. Our result may explain the underlying mechanism for increased betatrophin levels in obese or T2D subjects^17^,^18^,^19^,^29^ who are likely to have activated ER stress. Among the three essential pathways for unfolded protein response induced by ER stress, PERK-eIF2α-activating transcription factor 4 (ATF4) signaling regulates lipogenesis and hepatic steatosis by activating lipogenic genes, such as SREBP-1c^27^. We also confirmed that palmitate- or tunicamycin-treated HepG2 cells and the livers of various NAFLD mouse models had increased p-eIF2α and ATG6 levels, indicating the activation of ER stress. In addition, TLR signaling was involved in the induction of betatrophin expression in hepatocytes.

To date, all human studies have focused on investigating the relationship between serum betatrophin levels and diabetes, obesity, or dyslipidemia. An additional novel finding of this study is that we observed significantly increased betatrophin concentrations in subjects with NAFLD independent of diabetic status. However, this significant association between NAFLD and betatrophin levels was diminished when HOMA-IR was added in the final regression model, implying that insulin resistance was involved as a mediator in this association. Higher serum betatrophin levels were found in subjects with T1D^16^ and T2D^17^,^18^,^19^,^30^,^31^ whereas conflicting results were also reported in T2D^32^. Consistent with our data, several studies also reported that betatrophin levels were positively correlated to BMI^17^,^29^,^31^, fasting glucose^17^,^19^,^31^, HbA1c^18^,^19^, and estimated glomerular filtration rate (eGFR)^31^, and were negatively correlated to age^31^. Some studies showed a correlation between betatrophin concentration and TC^33^, LDL-C^33^, HDL-C^32^, and TG levels^32^. These discrepancies in the association of betatrophin levels with metabolic parameters in human studies may be explained by differences in patient populations. Further studies are required to elucidate this issue.

The current study has several strengths. Foremost, this translational research demonstrated that betatrophin levels were significantly increased in patients with NAFLD and in various fatty liver mouse models, implying that betatrophin may be a novel biomarker for NAFLD, similar to fetuin-A^34^ or selenoprotein P^35^. Additionally, we exposed one of the regulatory mechanisms involved in betatrophin expression in hepatocytes. Our study also has limitations, which can be addressed with further research. First, we measured serum betatrophin levels in a rather small number of subjects. Despite the relatively small sample size, the difference in betatrophin levels in the presence of NAFLD was significant, indicating a prominent association. Second, the cross-sectional study design does not permit unequivocal conclusions regarding causal relationships between betatrophin and NAFLD. Additional studies with genetically engineered mouse models, such as betatrophin knockout or betatrophin-expressing transgenic animals, should be conducted to elucidate the putative role of betatrophin in NAFLD. Tissue-level betatrophin expression and its relationship with hepatic lipid contents were not confirmed in the human liver specimens due to a lack of tissue samples. Finally, despite the clinical importance of discriminating NASH from NAFLD, we were unable to assess betatrophin’s ability to detect NASH as we lacked data regarding liver biopsies.

In conclusion, regardless of diabetic and obesity status, NAFLD subjects had significantly elevated circulating levels of betatrophin. The expression of betatrophin was highly induced by palmitate- or chemical-mediated ER stress in hepatocytes as well as in various fatty liver mouse models. This result indicates that betatrophin may be a candidate for use as a novel biomarker for NAFLD detection. Further research is warranted to investigate the functional role of betatrophin in NAFLD development and its mechanism of regulation.

## Methods

### Human subjects

We analyzed baseline clinical data and serum samples from subjects with NAFLD and T2D in the Severance metabOlic syndrome & NAFLD cohort (SONA cohort), an ongoing prospective cohort study supported by the Korea Health Industry Development Institute (KHIDI), for the cross-sectional study on betatrophin. A total of 164 subjects (30 normal glucose tolerant control subjects, 25 patients with IFG, and 109 patients with T2D) were recruited by the Diabetes Center at the tertiary-level, university-affiliated Severance Hospital, Yonsei University College of Medicine, from April 2014 to June 2015. Among them, 134 age- and sex-matched subjects were screened for having fatty liver by either abdominal ultrasound (n = 117, 87%) or computed tomography (CT, n = 17, 13%). Abdominal ultrasonography was performed with a 3.5-MHz transducer by trained radiologists who were blinded to the patients’ clinical and laboratory data, and fatty liver was defined based on standard criteria^36^. The liver attenuation index, derived from the difference between mean hepatic and splenic attenuation assessed from non-contrast CT images, was calculated for the diagnosis of fatty liver^37^. Control subjects were recruited after proper laboratory examination from individuals who visited the clinic for evaluating their health status due to their concern of having diabetes or other benign endocrinologic disorders such as benign thyroid nodules and osteopenia. The diagnoses of IFG and T2D were determined based on the American Diabetes Association guidelines^38^. Subjects who met the following criteria were excluded based on our protocol: (1) normal glucose tolerant subjects who took medications known to affect glucose or lipid metabolism; (2) types of diabetes other than T2D; (3) alcohol consumption >140 g/week for men and 70 g/week for women; (4) positive serologic markers for hepatitis B or hepatitis C virus; (5) presence of liver cirrhosis; and (6) (eGFR) <60 mL/min per 1.73 m^2^. Written informed consent was secured from all of the participants, and the protocol of this study was approved by the Institutional Review Board at Severance Hospital. All experiments were performed in accordance with relevant guidelines and regulations.

### Clinical and laboratory measurements

Body mass index (BMI) was calculated as weight in kilograms divided by the square of the height in meters. Alcohol consumption was examined by a detailed interview and analyzed as previously described^39^. Following overnight fasting >8 h, blood samples were drawn from the antecubital vein, serum was separated from blood specimens within 60 min of acquisition, and serum was stored at −70 °C until analysis at a later date. Serum levels of fasting and postprandial glucose, TC, TG, HDL-C, AST, and ALT were measured in a certified laboratory by standard methods. HbA1c was measured by high-performance liquid chromatography with a Variant II Turbo chromatograph (Bio-Rad Laboratories, Hercules, CA). Fasting serum insulin levels were determined by immunoradiometric assay (Beckman Coulter, Fullerton, CA). LDL-C was calculated using the Friedewald formula: LDL-C (mg/dL) = TC (mg/dL)−[HDL-C (mg/dL) + TG (mg/dL) /5]. The HOMA-IR^40^ was calculated as previously described.

Serum betatrophin levels were measured in duplicate using a commercially available human ELISA kit (Phoenix Pharmaceuticals, Burlingame, CA) according to the manufacturers’ instructions, as previously described in a detailed published report^17^,^31^. The sensitivity of the betatrophin ELISA was 0.01 μg/L, and inter- and intra-assay coefficients of variation were <15% and <10%, respectively. Immunoblotting was used to confirm whether the antibody used in the ELISA kit precisely detected betatrophin at the expected size of ~22.5 kDa from human serum samples and betatrophin protein.

### Cell culture and materials

HepG2 cells were maintained in high glucose Dulbecco’s modified Eagle’s medium (GE Healthcare Hyclone, Sungnam, Korea), supplemented with 10% fetal bovine serum, 100 U penicillin, and 100 μg streptomycin at 37 °C in a humidified incubator containing 5% CO_2_ and 95% air. Cells were treated with various concentrations of tunicamycin (ranging from 0.1 to 3 μg/ml; #654380, Calbiochem, La Jolla, CA) for 12 h prior to performing quantitative real-time polymerase chain reaction (PCR) and for 24 h prior to performing immunoblots. For the time-dependent experiment (from 0 to 24 h), 1 μg/ml of tunicamycin was added to the HepG2 cells. Palmitate (cat#P9767, Sigma-Aldrich, St. Louis, MO) was used after conjugation with bovine serum albumin (5%) and 4-PBA, 3 mM, chemical chaperone, cat#SML0309, Sigma-Aldrich) was pretreated for 1 h prior to adding either tunicamycin or palmitate. LPS (cat#L6529, Sigma-Aldrich) was used as indicated.

### RNA isolation and real-time PCR

Total RNA was extracted using TRIzol^®^ reagent (Invitrogen) according to the manufacturer’s instructions and subjected to reverse transcription using the High Capacity cDNA Transcription kit (Applied Biosystems, Foster City, CA) followed by quantitative real-time PCR using an ABI 7500 sequence detection system (Applied Biosystems). PCR was performed using the following primers (for SYBR Green): human betatrophin, forward (5′-gag act cag atg gag gag ga-3′) and reverse (5′-atg ctg ctg tgc cac cat ct-3′); mouse betatrophin, forward (5′-gct tta cac ctt cga gct ga-3′) and reverse (5′-atc cag gta gtc tca ggc tg-3′); TNFα, forward (5′-ctg tag ccc acg tcg tag c-3′) and reverse (5′-ttg aga tcc atg ccg ttg-3′); COL1A1, forward (5′-cct ggt aaa gat ggt gcc-3′) and reverse (5′-cac cag gtt cac ctt tcg cac c-3′); TGFβ, forward (5′-tga cgt cac tgg agt tgt acg g-3′) and reverse (5′-ggt tca tgt cat gga tgg tgc-3′); GAPDH, forward (5′-aac ttt ggc att gtg gaa gg-3′) and reverse (5′-tgt tcc tac ccc caa tgt gt-3′); β-actin, forward (5′-gga ctt cga gca aga gat gg-3′) and reverse (5′-agc act gtg ttg gcg tac ag-3′). Quantitative analyses were conducted using the ΔΔcycle threshold method and StepOne Software version 2.2.2.

### Protein extraction and immunoblotting

Mouse livers and HepG2 cells were lysed in RIPA buffer (Cell Signaling Technology, Danvers, MA), and the protein extract was measured using the Bradford assay (Bio-Rad, 162-0115, Hercules, CA). Equal amounts of proteins (50 μg) were heat-denatured in 4× sample buffer (2% sodium dodecyl sulfate, 62.5 mM Tris (pH 6.8), 0.01% bromophenol blue, 1.43 mM mercaptoethanol, and 0.1% glycerol), separated on 10% or 12% sodium dodecyl sulfate-polyacrylamide gels, and electroblotted onto a nitrocellulose membrane (Bio-Rad). Membranes were subsequently treated with the appropriate antibodies against the following proteins: betatrophin (cat#TA326696, OriGene Technologies, Rockville, MD), phosphorylated eukaryotic translation initiation factor 2α (p-eIF2α; cat#ab32157, Abcam, Cambridge, MA), Activating transcription factor 6 (ATF6; cat# MAB0082, Abnova, Taipei city, Taiwan), NF-κB (p65, cat#sc-372, Santa Cruz Biotechnology, Santa Cruz, CA) and β-actin (cat#a5441, Sigma-Aldrich).

### Animals

Eight-week-old db/db male mice (n = 6, C57BL/6J background), ob/ob male mice (n = 6, C57BL/6J background, Jackson Laboratory, 000632), and normal C57BL/6J mice (n = 24, Jackson Laboratory, 000664), purchased from Orient Bio (Sungnam, Korea), were maintained at ambient temperature (22 ± 1 °C) with 12-h light-dark cycles and free access to water and food. Db/db and ob/ob mice were fed a normal chow diet (Dyets, Inc., Bethlehem, PA) for 4 weeks. For the HFD-induced obesity and methionine-choline deficient (MCD) diet-induced fatty liver models, C57BL/6J mice were randomly divided into three groups: a control group was fed a chow diet for 4 weeks (n = 6) and 8 weeks (n = 6), an HFD group (n = 6) was fed a HFD (60% kcal from fat, Research diet, Bethlehem, PA) for 8 weeks, and an MCD group (n = 6) was fed an MCD diet (Research diet) for 4 weeks. Mouse body weight, blood glucose levels, and food intake were measured three times a week over the experimental period. Animals were sacrificed after a 6-h fast. Livers were isolated, immediately freeze-clamped in liquid nitrogen, and stored at −80 °C until assays were conducted. For *in vivo* experiments with tunicamycin, C57BL/6J mice (n = 4 to 6 per each group) were given a single 1 mg/kg body weight intraperitoneal injection of tunicamycin and then were sacrificed after 24 h. One hundred mg/kg 4-PBA was pre-treated in mice by oral gavage before injection of tunicamycin. All experimental procedures performed in this study followed ethical guidelines for animal studies and were approved by the Institutional Animal Care and Use Committee of Yonsei University College of Medicine (IACUC No. 2013-0147-1). All experiments were performed in accordance with relevant guidelines and regulations.

### Hepatic triglyceride measurement

Following liver homogenization, TG content in liver tissues was measured with the Triglycerides Quantification Kit (Biovision, K622, Milipitas, CA) according to the manufacturer’s instructions.

### Statistical analysis

Data were compared using the Student’s t-test, analysis of variance, or Pearson’s χ^2^ test as appropriate followed by *posthoc* Bonferroni tests, using the SPSS 20.0 program (SPSS Institute, Chicago, IL). Mediation effect of HOMA-IR was examined by Baron Kenny Procedure^41^ using R version 3.2.2 (The R Foundation for Statistical Computing, Vienna, Austria)^42^. Graphs were plotted using PRISM (Version 5.0, GraphPad Software Inc, San Diego, CA). For the human study, data were presented as mean ± SD or N (%). Fasting glucose, AST, ALT, TG, fasting insulin, and HOMA-IR levels were log transformed for the analyses, because the value distributions were skewed based on the Kolmogorov-Smirnov test. We conducted Spearman correlation analyses to examine the relationship between serum betatrophin levels and clinical and biochemical variables, followed by multivariate regression analyses with adjustment for various covariates. Model 1 included variables that showed statistical significance in the simple correlation analysis except AST, ALT, and insulin resistance-related indices. Model 2 adjusted for Model 1 + AST and ALT, and Model 3 adjusted for Model 2 + HOMA-IR (fasting glucose was removed due to collinearity with HOMA-IR). The ability of serum betatrophin levels to predict NAFLD was evaluated by ROC curves and the AUROC was calculated. A P-value of <0.05 was considered statistically significant. All results are presented as representative data from six to nine experiments.

## Additional Information

**How to cite this article**: Lee, Y.-h. *et al.* Association between betatrophin/ANGPTL8 and non-alcoholic fatty liver disease: animal and human studies. *Sci. Rep.* **6**, 24013; doi: 10.1038/srep24013 (2016).

## Figures and Tables

**Table 1:** Clinical and biochemical characteristics of the study population according to non-alcoholic fatty liver disease (NAFLD) status.

	No NAFLD (N = 38)	NAFLD (N = 96)	P
Age (years)	56.3 ± 10.4	52.4 ± 12.8	0.094
Sex (Female%)	53	41	0.207
BMI (kg/m^2^)	23.4 ± 3.3	26.7 ± 4.1	<0.001
NL/overweight/obese (%)	40/30/30	16/23/61	0.002
SBP (mmHg)	121.8 ± 13.7	124.4 ± 13.7	0.321
DBP (mmHg)	74.6 ± 10.3	77.3 ± 11.3	0.203
NL/IFG/T2D (%)	32/16/52	9/12/79	0.003
Fasting glucose (mg/dl)*	113.1 ± 26.8	133.5 ± 35.8	0.002
Postprandial glucose (mg/dl)	164.8 ± 63.0	210.9 ± 115.2	0.110
HbA1c (%)*	6.5 ± 0.8	7.0 ± 1.0	0.007
HbA1c (mmol/mol)*	47.3 ± 8.7	53.4 ± 11.3	0.007
AST (U/l)*	18.7 ± 5.9	27.4 ± 16.2	<0.001
ALT (U/l)*	16.7 ± 6.3	34.4 ± 26.2	<0.001
TC (mg/dl)	186.8 ± 32.2	176.5 ± 46.8	0.152
LDL-C (mg/dl)	109.3 ± 32.2	100.9 ± 36.2	0.242
TG (mg/dl)*	109.0 ± 60.4	171.6 ± 145.0	0.003
HDL-C (mg/dl)*	53.0 ± 15.3	44.4 ± 10.7	0.005
Fasting insulin (μU/mL)*	6.5 ± 4.0	12.2 ± 9.7	<0.001
HOMA-IR*	1.9 ± 1.2	4.2 ± 3.7	<0.001
Medication			
Metformin (%)	28	52	0.021
DPP4 inhibitors (%)	19	28	0.328
SU (%)	9	19	0.217
Insulin analogues (%)	3	5	0.640

^*^Log-transformed. NL, normal; SBP, systolic blood pressure; DBP, diastolic blood pressure; IFG, impaired fasting glucose; T2D, type 2 diabetes; AST, aspartate transaminase; ALT, alanine aminotransferase; TC, total cholesterol; TG, triglyceride; DPP4, dipeptidyl peptidase-4; SU, sulfonylurea.

**Table 2:** Spearman correlation and multivariate regression analyses of clinical and biochemical parameters associated with serum betatrophin levels.

	Spearman correlation		Model 1		Model 2		Model 3	
	*ρ*	P	STD *β*	P	STD *β*	P	STD *β*	P
Age (years)	**−0.199**	**0.021**	**−**0.130	0.170	**−**0.120	0.222	**−**0.128	0.252
Sex	−0.052	0.552	0.075	0.419	0.077	0.418	0.083	0.420
BMI (kg/m^2^)	**0.281**	**0.001**	**−**0.010	0.916	**−**0.026	0.792	0.035	0.751
SBP (mmHg)	0.056	0.526	–	–	–	–	–	–
DBP (mmHg)	**0.219**	**0.012**	0.084	0.380	0.085	0.377	0.165	0.122
Fasting glucose (mg/dl)*	**0.310**	**<0.001**	**−**0.189	0.213	**−**0.192	0.220	–	–
Postprandial glucose(mg/dl)	**−**0.002	0.988	–	–	–	–	–	–
HbA1c (%)	**0.439**	**<**0.001	**0.410**	**0.008**	**0.398**	**0.014**	**0.300**	**0.005**
AST (U/l)*	**0.226**	**0.009**	–	–	-0.012	0.944	0.062	0.724
ALT (U/l)*	**0.326**	**<0.001**	–	–	0.072	0.690	**−**0.036	0.855
TC (mg/dl)	0.047	0.594	–	–	–	–	–	–
LDL-C (mg/dl)	0.051	0.569	–	–	–	–	–	–
TG (mg/dl)*	**0.241**	**0.006**	0.117	0.212	0.102	0.690	0.094	0.385
HDL-C (mg/dl)*	**−**0.008	0.929	–	–	–	–	–	–
Fasting insulin (μU/mL)*	**0.255**	**0.009**	–	–	–	–	–	–
HOMA-IR	**0.320**	**<0.001**	–	–	–	–	0.008	0.949
NAFLD	**0.330**	**<0.001**	**0.204**	**0.028**	**0.195**	**0.040**	0.089	0.391

^*^Log-transformed. SBP, systolic blood pressure; DBP, diastolic blood pressure; AST, aspartate transaminase; ALT, alanine aminotransferase; TC, total cholesterol; TG, triglyceride; NAFLD, non-alcoholic fatty liver disease. Model 1 adjusted for variables which had significant simple correlation with betatrophin levels. Model 2 adjusted for Model 1 + AST, ALT and, Model 3 adjusted for Model 2 + HOMA-IR. Values with significance are printed in bold.
